# Single-Nucleus RNA-seq of Normal-Appearing Brain Regions in Relapsing-Remitting vs. Secondary Progressive Multiple Sclerosis: Implications for the Efficacy of Fingolimod

**DOI:** 10.3389/fncel.2022.918041

**Published:** 2022-06-17

**Authors:** Yasuyuki Kihara, Yunjiao Zhu, Deepa Jonnalagadda, William Romanow, Carter Palmer, Benjamin Siddoway, Richard Rivera, Ranjan Dutta, Bruce D. Trapp, Jerold Chun

**Affiliations:** ^1^Translational Neuroscience Initiative, Sanford Burnham Prebys Medical Discovery Institute, La Jolla, CA, United States; ^2^Biomedical Sciences Graduate Program, School of Medicine, University of California, San Diego, La Jolla, CA, United States; ^3^Department of Neurosciences, Lerner Research Institute, Cleveland Clinic, Cleveland, OH, United States

**Keywords:** neuroinflammation, S1P_1_, FTY720, siponimod, ozanimod, ponesimod, lysophospholipid receptors

## Abstract

Multiple sclerosis (MS) is an immune-mediated demyelinating disease that alters central nervous system (CNS) functions. Relapsing-remitting MS (RRMS) is the most common form, which can transform into secondary-progressive MS (SPMS) that is associated with progressive neurodegeneration. Single-nucleus RNA sequencing (snRNA-seq) of MS lesions identified disease-related transcriptomic alterations; however, their relationship to non-lesioned MS brain regions has not been reported and which could identify prodromal or other disease susceptibility signatures. Here, snRNA-seq was used to generate high-quality RRMS vs. SPMS datasets of 33,197 nuclei from 8 normal-appearing MS brains, which revealed divergent cell type-specific changes. Notably, SPMS brains downregulated astrocytic sphingosine kinases (*SPHK1/2*) – the enzymes required to phosphorylate and activate the MS drug, fingolimod. This reduction was modeled with astrocyte-specific *Sphk1/2* null mice in which fingolimod lost activity, supporting functionality of observed transcriptomic changes. These data provide an initial resource for studies of single cells from non-lesioned RRMS and SPMS brains.

## Introduction

Single cell transcriptomics has become a principal gateway to understand cellular states and characteristics in normal and diseased tissues (Regev et al., 2017; Lahnemann et al., 2020). Approaches for analyzing single brain cells by single-nucleus RNA sequencing (snRNA-seq) (Gole et al., 2013; Fan et al., 2016; Lake et al., 2016, 2017, 2018; Zheng et al., 2017) have successfully generated reference datasets from non-diseased and diseased human brains that include Alzheimer’s disease (Mathys et al., 2019), Parkinson’s disease (Aneichyk et al., 2018), amyotrophic lateral sclerosis (Maniatis et al., 2019) and multiple sclerosis (MS) (Beltran et al., 2019; Jakel et al., 2019; Masuda et al., 2019; Schirmer et al., 2019).

MS is a neuroinflammatory disease that produces neurodegeneration. It is characterized pathologically by inflammation and demyelination that produce white matter plaque lesions and neuronal damage/loss in the central nervous system (CNS) (Hickey, 1999; Thompson et al., 2018). Relapsing-remitting MS (RRMS) is the most common disease course, which can transition into secondary progressive MS (SPMS) that is characterized by progressive neurological disability (Hickey, 1999; Thompson et al., 2018). Pathophysiological differences have been reported between RRMS vs. progressive forms of MS, contrasting with subtypes of progressive MS – primary progressive MS (PPMS) and SPMS – that share similar neuropathologies (Lassmann, 2018).

MS lesions from patient samples that have been analyzed by snRNA-seq thus far have generally been compared to non-diseased brains. Reported features of RRMS include loss of excitatory neurons in the upper-cortical layers, and gene expression profiles characteristic of stressed oligodendrocytes (OLs), reactive astrocytes, and activated microglia (Schirmer et al., 2019). In SPMS lesions, there is oligodendroglial heterogeneity, with reduced numbers of oligodendrocyte progenitor cells (OPCs) and loss of *OPALIN*^+^ oligodendrocyte sub-populations (Jakel et al., 2019). Another study that compared nuclei isolated from tumefactive MS lesions highlighted differences between activated microglia vs. healthy microglia of non-diseased controls (Masuda et al., 2019). These snRNA-seq studies comparing changes between MS brain lesions vs. control brains focused preferentially on cells within and around lesions that lacked comparable cellular controls, as indicated by markedly different cell populations revealed by t-distributed stochastic neighbor embedding (tSNE; Jamieson et al., 2010) or uniform manifold approximation and projection (UMAP; Becht et al., 2019) clustering plots.

The possibility that there are disease-related transcriptomic changes in the MS brain apart from demyelinating lesions is predicted by the known discordance between lesion burden and clinical presentation, as well as gray matter changes (Geurts et al., 2012) and brain volume loss in MS (Bermel and Bakshi, 2006). To identify possible global changes in disease-validated RRMS and SPMS brains, neuroanatomically matched, normal-appearing prefrontal cortices with no apparent MS lesions were assessed by snRNA-seq. Results from these studies could provide insights into therapeutic mechanisms, and we investigated one such link involving sphingosine kinases and the loss of fingolimod efficacy in patients with progressive MS.

## Materials and Methods

### Human Brain Tissues

Postmortem prefrontal cortices (BA10) from RRMS and SPMS donors were collected, frozen immediately, and stored at −80°C at the Cleveland Clinic and the guidelines of the Cleveland Clinic Human Research Ethics Committee were followed (Dutta et al., 2019). The detailed sample information is provided in Supplementary Table 1.

### Nuclear Isolation, 10× Genomics Platform, and Short-Read Sequencing

Tissues were cut on a cryostat (Leica) and a total of 300 μm sections from each were stored at −80°C until use. The buffers used in this procedure were made in autoclaved diethyl pyrocarbonate (DEPC; MilliporeSigma, Cat # D5758)-treated water. All the steps from here on were performed at 4°C with additional equilibration time at each step. The samples were initially placed in 0.5 mL nuclear extraction buffer [NEB; 0.32 M Sucrose, 5 mM CaCl_2_.2H_2_0, 3 mM (CH_3_COO)_2_Mg.4H_2_O, 0.1 mM EDTA, 10 mM Tris/HCl (pH 8.0), 0.1% Triton X-100 with cOmplete*^rmTM^* mini protease inhibitor cocktail tablet (MilliporeSigma, Cat # 11836153001), and fresh 0.2% RNase inhibitor (Takara Bio, Cat # 2313A)] for 15 min. After adding another 0.5 mL NEB, the samples were homogenized thoroughly with a Dounce homogenizer. Upon transferring this homogenate to a new tube, the Dounce homogenizer was rinsed out with 1 mL of NEB. Homogenates were then passed through a CellTrics^®^ 50 μm filter (Sysmex, Cat # 04-004-2324). After washing the filter with NEB once, the filtrates were centrifuged at 1600 × *g* for 5 min. The resulting pellets were resuspended with 1 mL of PBSE (1× PBS, 2 mM EGTA and fresh 0.2% RNase inhibitor), transferred into a new tube, diluted with PBSE, and centrifuged again at 1600 × *g* for 5 min. Meanwhile, density gradients of OptiPrep™ (MilliporeSigma, Cat # D1556) were made in FACS tubes with 0.4 mL 35% OptiPrep™ on the bottom and 2 mL 10% OptiPrep™ on the top. Solutions used for density gradients were as follows: OptiPrep™, solution B (6× concentrations: 120 mM Tricine/NaOH pH 7.8, 150 mM KCl, and 30 mM MgCl_2_.6H_2_O) and solution D (0.25 M Sucrose in 1× solution B with freshly added 0.2% RNase inhibitor). The OptiPrep™ solutions were diluted with solution D to make the final concentrations to 35 and 10%. Nuclear pellets were resuspended with 0.2 mL solution D, followed by addition of 0.2 mL of 10% OptiPrep solutions, which were layered onto the 10% OptiPrep™ solution, and centrifuged at 3250 × *g* for 10 min. The nuclei that settled at the boundary of 35 and 10% were collected, washed twice with PBSE containing 1% fatty acid-free bovine serum albumin (BSA; Gemini Bio, Cat # 700-107P), passed through a CellTrics 50 μm filer, washed again with PBSE + BSA, and labeled with DAPI (1:100,000, 4′,6-diamidino-2-phenylindole; MilliporeSigma, Cat # D9542). About 3 × 10^5^ nuclei were sorted by a BD FACSAria Fusion (BD Biosciences) into a collection tube that was precoated and filled with 1 mL of PBSE + BSA. The nuclei were washed once and resuspended with PBSE + BSA to obtain around 1500 cells/μL, and the counts were confirmed by Countess II (Thermo Fisher Scientific). Single-nucleus capture (target capture of 5000 nuclei per sample) and library preparation was conducted using Chromium Next GEM Single Cell 3′ GEM Library and Gel Bead Kit v3 (10× Genomics, Cat # PN-1000075) and Chromium Single Cell B Chip Kit (10× Genomics, Cat # PN-1000074) according to the manufacturer’s instructions without modification. Single-nucleus libraries were sequenced on the Illumina HiSeq 3000 machine at GENEWIZ, Inc. (United States).

### Bioinformatic Analyses and Statistical Methods

Raw FASTQ files were input into the Cell Ranger count pipeline (Cell Ranger V3.0.2, 10× Genomics) to align reads to the GRCh38 human genome, quality filter cellular barcodes and unique molecular identifiers (UMIs), and count UMIs by gene. To account for unspliced pre-mRNA transcripts in the nucleus, a custom pre-mRNA annotation file was generated and supplied to Cell Ranger as per 10× Genomics instructions. Gene-UMI count matrices generated from individual samples were filtered to exclude genes detected in less than three nuclei and to retain nuclei with at least 300 genes expressed. Next, dying cells and multiplets were removed by excluding nuclei with >1% mitochondrial count fraction or transcript number exceeding the 75th quantile + 1.5 × IQR (Interquartile Range). After these stringent filtering steps, we obtained a total of 33,197 high quality nuclei from 8 MS brains (3 RRMS vs. 5 SPMS). After this initial quality filtering, UMI raw count matrices from individual samples were normalized using a regularized negative binomial regression method (Hafemeister and Satija, 2019) to regress out mitochondrial count fractions and then were integrated into one combined dataset using Seurat V3.0 (Stuart et al., 2019) in R. Next, nuclei in the integrated dataset were clustered through a shared nearest neighbor (SNN) algorithm and visualized in two-dimensional space using UMAP (Becht et al., 2019). Cell types were annotated by performing cell label transfer in Seurat based on a reference normal brain snRNA-seq dataset that we previously published (Lake et al., 2018), which produced cell type-specific markers unbiasedly (Supplementary Figure 1 and Supplementary Table 3). Differentially expressed genes [DEGs; FDR-adjusted *p* < 0.05, log_2_(fold change) > 1.1] in each cluster were identified by fitting a hurdle model adjusting for sex, age, and RNA integrity number (RIN) scores in MAST (Finak et al., 2015). Cell detection rates (CDRs) were calculated from a transformed UMI count matrix that has undergone a binary transformation based on presence-or-absence of gene expression. The statistical difference of CDR between RRMS and SPMS groups was determined by the non-parametric Wilcoxon rank-sum test. The vastly altered genes (VAGs) were defined as the intersection between DEGs and genes with significant CDRs. Pathway analyses were executed in the Reactome Pathway Database^1^ and the results are provided in Supplementary Tables 7–9. FASTAQ files have been deposited into the Gene Expression Omnibus (GEO^2^). The accession number is GSE179590.

### Animal Studies

Animal protocols were approved by the Institutional Animal Care and Use Committee of the Sanford Burnham Prebys Medical Discovery Institute and conformed to National Institutes of Health guidelines and public law. The *Sphk1/2^flox/flox^* mice (Pappu et al., 2007) were crossed with human GFAP-Cre [FVB-Tg(GFAP-cre)25Mes/J, Cat# JAX:004600, RRID:IMSR_JAX:004600] mice to generate astrocyte specific *Sphk1/2* conditional KO (SK1/2-AsCKO: Sphk1/2*^flox/flox^*:GFAP-Cre) mice. Experimental autoimmune encephalomyelitis (EAE) was induced in 8- to 12-week-old female mice as previously described (Kihara et al., 2009, 2015a). Briefly, mice were immunized with 100 μL emulsions containing 150 μg myelin oligodendrocyte glycoprotein 35–55 (MOG35–55) (MEVGWYRSPFSRVVHLYRNGK, EZBiolab) in PBS and a mixture of Difco incomplete Freund’s adjuvant (BD Biosciences, Cat # 263910) and 4 mg/mL Difco Adjuvant (*Mycobacterium tuberculosis* H37; BD Biosciences, Cat # 231141). Daily clinical scores were given as follows: 0, no sign; 0.5, mild loss of tail tone; 1.0, complete loss of tail tone; 1.5, mildly impaired righting reflex; 2.0, abnormal gait and/or impaired righting reflex; 2.5, hindlimb paresis; 3.0, hindlimb paralysis; 3.5, hindlimb paralysis with hind body paresis; 4.0, hind- and forelimb paralysis; and 4.5, death or severity necessitating euthanasia. Fingolimod was administered *via* gavage (1 mg/kg; gifted from Novartis). Although the clinical score is categorized as an ordinal scale, parametric test (two-way ANOVA) was used to determine statistical differences because our datasets distributed normally (*p* = 0.2 by Shapio–Wilk normality test). Use of parametric tests in ordinal scale (Norman, 2010; Sullivan and Artino, 2013; Awan and Dako, 2018) and statistical tests for EAE studies (Fleming et al., 2005; Baker and Amor, 2012; Baker et al., 2014) have been discussed elsewhere. Sphingosine1-phosphate (S1P) levels were quantified at the Center for Metabolomics and Mass Spectrometry at The Scripps Research Institute, United States.

## Results

### Single-Nucleus RNA Sequencing Identified Major Central Nervous System Cell Types From Non-lesioned Multiple Sclerosis Brains

**FIGURE 1:**
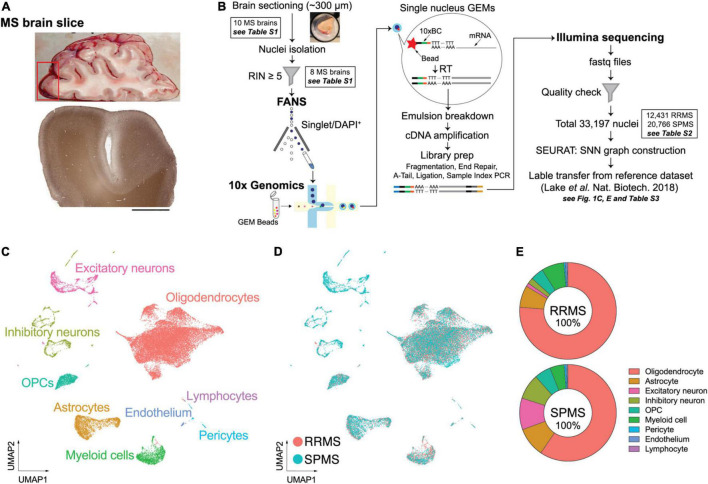
Experimental approach of snRNA-seq and unbiased cellular identification in MS brains. **(A)** Macroscopic and standard immunohistochemical labeling for myelin-specific proteolipid protein (PLP) of a representative MS brain. **(B)** Experimental workflow. Nuclei were isolated from ∼300 μm frozen MS brain sections by fluorescence-activated nuclear sorting (FANS) based on DAPI (4′,6-diamidino-2-phenylindole) positivity. Brain samples with a RNA integrity number (RIN) ≥5 were processed for 10× Genomics and snRNA-seq. **(C)** Unbiased cellular identification. Quality-control filtering identified a total of 33,197 nuclei from both RRMS and SPMS brains; these cells were then clustered using the shared nearest neighbor (SNN) clustering algorithm in the SEURAT package. The cell types were annotated based on the reference dataset of normal human brains (Lake et al., 2018) and are presented as a UMAP (uniform manifold approximation and projection) plot. Colors indicate specific cell clusters identified by SEURAT V.3.0. **(D)** UMAP plots for RRMS and SPMS cells. **(E)** Relative proportions of the nine cell types analyzed in the RRMS and SPMS brains (see details in Supplementary Table 3).

A previously developed snRNA-seq pipeline for human postmortem brains was used to assess gene expression signatures in normal-appearing MS brains. None of the 10 collected MS prefrontal cortices (Brodmann area 10; 5 from RRMS and 5 from SPMS) showed detectable demyelination (Figure 1A). No statistical differences in age, sex, or postmortem interval (PMI) were identified between RRMS vs. SPMS. The median Expanded Disability Status Scale (EDSS) was higher in SPMS than in RRMS (median EDSS = 9.0 vs. 4.0, respectively, Supplementary Table 1). Brain samples with an RIN ≥5 (median RIN = 7.35: Supplementary Table 1) were sectioned at ∼300 μm thickness and processed for fluorescence-activated nuclear sorting (FANS). DAPI^+^ singlet nuclei were barcoded using a 10× Genomics Chromium single cell 3′ reagent kit, followed by short-read sequencing on an Illumina HiSeq 3000 instrument (Figure 1B). Quality control filtering resulted in a total of 33,197 nuclei (12,431 nuclei from 3 RRMS brains vs. 20,766 nuclei from 5 SPMS brains; Supplementary Table 2). Small sample size and biological variability may limit the universality of the results, requiring analysis of larger sample sizes in the future. Cells were clustered using the Seurat SNN (SNN) algorithm and visualized as UMAP plots (Figure 1C).

Cell types were determined by label transfer from a reference dataset (Lake et al., 2018), resulting in nine major cell types including astrocytes, endothelial cells, excitatory and inhibitory neurons, OLs, OPCs, lymphocytes, myeloid cells (microglia/macrophages), and pericytes (Figure 1C). The reference dataset enabled cell type annotation but it was not used for gene expression comparison to avoid uncertain outcomes derived from different experimental techniques and conditions. Cell types were confirmed by the expression patterns of well-established marker genes (Supplementary Figure 1). UMAP plots showed consistent and largely overlapping layouts between RRMS vs. SPMS (Figures 1D,E and Supplementary Table 3). This indicated that gene expression in each cell type can be compared between MS forms. The results are presented in the following sections for neurons, OLs and astrocytes. Analyses of other cell types are available in Supplementary Figure 2. Inhibitory neurons were characterized by limited transcriptomic alterations (Supplementary Table 6), and therefore analyses centered on more affected cell types.

### Relapsing-Remitting MS Shows Lower Expression of Excitatory Neuronal Markers Than Secondary-Progressive MS

**FIGURE 2:**
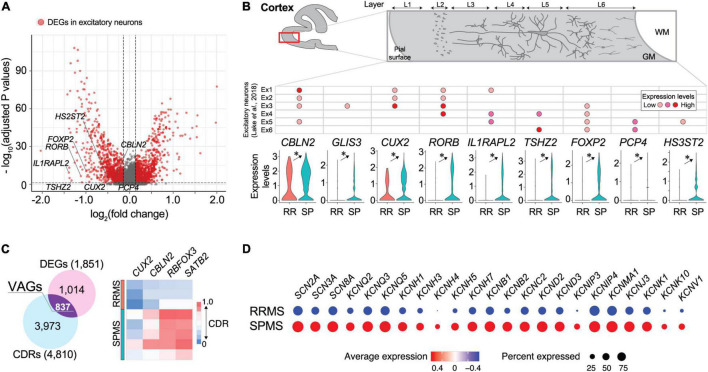
Transcriptomic divergence in neuronal populations between RRMS vs. SPMS. **(A)** Volcano plots of genes identified in excitatory neurons. *Red dots* represent differentially expressed genes (DEGs; adjusted *p*-values < 0.05 and fold change > 1.1) and *gray dots* represent non-significant genes. **(B)** Violin plots of excitatory neuron marker gene expression levels and a table for their expression patterns in the cortical layer determined by previous study (Lake et al., 2018). *, adjusted *p*-value < 0.05. **(C)** Venn diagram of DEGs and genes of differential CDRs (cell detection rates; the proportion of cells that express particular genes within each cell cluster), and a heatmap of excitatory neuron marker genes found in VAGs (vastly altered genes). **(D)** Dot plots of expression levels of sodium and potassium channel genes. Size and color indicate CDRs and expression levels, respectively. Statistical data for DEGs, CDRs, and VAGs are provided in Supplementary Tables 4–6.

Comparisons of gene expression profiles between the RRMS vs. SPMS excitatory neurons (RRMS 1.1%; SPMS 9.5% of total) identified 1851 DEGs (Figure 2A and Supplementary Table 4). Marker genes for excitatory neurons (*CBLN2*, *GLIS3*, *CUX2*, *RORB*, *IL1RAPL2*, *TSHZ2*, *FOXP2*, *PCP4*, *HS3ST2*) were significantly downregulated in RRMS compared to SPMS (Figure 2B). In addition to DEGs, cell detection rates (CDRs) were calculated as the proportion of cells that express particular genes within each cell cluster (Supplementary Table 5). The CDRs in combination with DEGs enabled the identification of VAGs that represented alterations in both expression level and frequency (Supplementary Table 6). These analyses identified 837 VAGs in excitatory neurons that included upper layer marker genes (*CUX2*, *CBLN2*, *RBFOX3*, *SATB2*) whose CDRs were decreased in RRMS as compared to SPMS (Figure 2C and Supplementary Table 6). Reactome pathway analyses of upregulated DEGs in RRMS revealed enrichment of the immune response-related pathways (e.g., antigen presentation, interferon pathways: Supplementary Table 7). The neuronal loss of RRMS brains, which might reflect prior loss of susceptible RRMS neurons in SPMS, may produce neural circuit rewiring issues during disease progression, resulting in cognitive impairment in SPMS (Brochet and Ruet, 2019; Benedict et al., 2020). In SPMS, excitatory neurons showed enrichment of synaptic transmission pathways (e.g., neurexins/neuroligins, channels, axon guidance: Supplementary Table 7). Notably, DEGs associated with sodium and potassium channels (Figure 2D) were highly upregulated in SPMS, which might reflect potential channelopathy-like defects in SPMS (Kumar et al., 2016; Roostaei et al., 2016; Zhong et al., 2016) because impairment of channels causes axonal degeneration (Dutta and Trapp, 2014).

### Secondary-Progressive MS Shows Decreased Expression of Oligodendrocyte Markers Compared to Relapsing-Remitting MS

**FIGURE 3:**
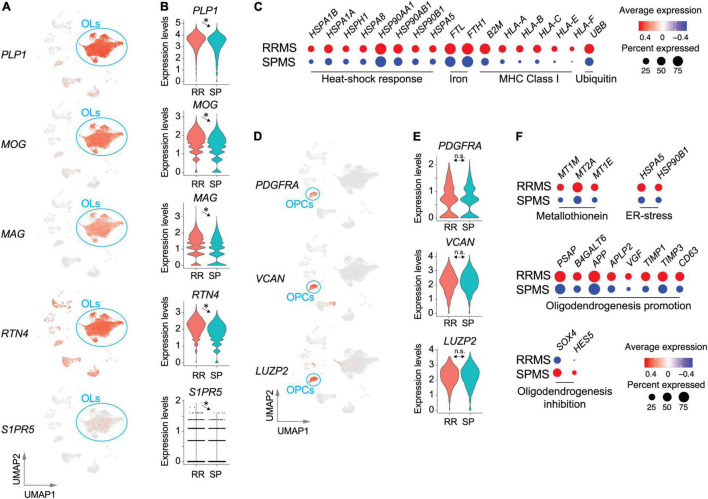
Transcriptomic pathological signatures of MS oligodendrocytes (OLs) and oligodendrocyte progenitor cells (OPCs). UMAP **(A)** and violin plots **(B)** of OL marker genes. *, adjusted *p*-value < 0.05. **(C)** Dot plots for genes associated with heat-shock response, iron accumulation, major histocompatibility complex class I, and ubiquitin-mediated protein degradation. Size and color indicate the CDRs and expression levels, respectively. UMAP **(D)** and violin plots **(E)** of OPC marker genes. n.s., non-significant. **(F)** Dot plots of genes associated with metallothionein pathway genes, ER-stress related genes, and oligodendrogenesis-promoting or inhibiting genes. Size and color indicate frequency and expression levels, respectively. Statistical results for DEGs, CDRs, and VAGs are provided in Supplementary Tables 4–6.

Most identified cells were OLs in both RRMS and SPMS samples (∼70% of total). OLs were characterized by the expression of *PLP1*, *MOG*, and *MAG* (Figures 3A,B and Supplementary Figure 1). These genes, as well as *RTN4* (encoding Nogo-A) and *S1PR5* (Figures 3A,B), were significantly higher in RRMS vs. SPMS. OL depletion was not observed in these normal-appearing samples, contrasting with their loss from MS lesions (Jakel et al., 2019), while lower expression levels of OL marker genes in SPMS suggested a potential vulnerability of OLs (Dutta and Trapp, 2014), which may lead to OL loss in and around developing active SPMS lesions. OLs showed the third-highest number of DEGs (1145), ∼94% of which were markedly upregulated in RRMS vs. SPMS (Supplementary Table 4) and included genes associated with heat-shock response (*HSPA1B*, *HSPA1A*, *HSPH1*, *HSPA8*, *HSP90AA1*, *HSP90AB1*, *HSP90B1*, and *HSPA5*), iron accumulation (*FTL* and *FTH1*), major histocompatibility complex class I (*B2M*, *HLA-A*, *HLA-B*, *HLA-C*, *HLA-E*, and *HLA-F*), and ubiquitin-mediated protein degradation (*UBB*) (Figure 3C). Reactome pathway analyses of the extracted 158 VAGs (Supplementary Tables 5, 6) highlighted the nonsense-mediated mRNA decay (NMD) pathway that functions in RNA surveillance to degrade aberrant mRNAs (Supplementary Table 8). The expression of NMD antagonist *UPF3A* in RRMS OLs was elevated as compared to SPMS, indicating an accumulation of aberrant RNAs produced by low NMD in RRMS OLs (Shum et al., 2016). Collectively, RRMS OLs might reflect more severe cellular stress, leading to subsequent OL loss in SPMS.

OPCs (RRMS 4.6%; SPMS 5.8% of total) characterized by expression of *PDGFRA*, *VCAN*, and *LUZP2* (Figures 3D,E and Supplementary Figure 1) presented 243 DEGs (Supplementary Table 4), 34 of which were extracted as VAGs (Supplementary Figure 3). Reactome pathway analyses identified upregulation of metallothionein pathway genes (*MT1M*, *MT2A*, and *MT1E*) and ER-stress related genes (*HSPA5* and *HSP90B1*) in RRMS (Figure 3F and Supplementary Table 9). Importantly, elevation of myelin formation-promoting genes (*PSAP*, *B4GALT6*, *APP*, *APLP2*, *VGF*, *TIMP1*, *TIMP3*, *CD63*) (Hiraiwa et al., 1999; Alvarez-Saavedra et al., 2016; Yoshihara et al., 2018; Nicaise et al., 2019; Truong et al., 2019) were accompanied by decreases in oligodendrogenesis-inhibiting genes (*SOX4* and *HES5*) (Braccioli et al., 2018) in RRMS OPCs, as compared to SPMS OPCs (Figure 3F). These results suggest that OPC maturation and myelination were more active in RRMS brains than SPMS brains.

### Relapsing-Remitting MS Shows Higher Expression of Pan-Reactive Astrocyte Markers and Immediate-Early Genes Compared to Secondary-Progressive MS

**FIGURE 4:**
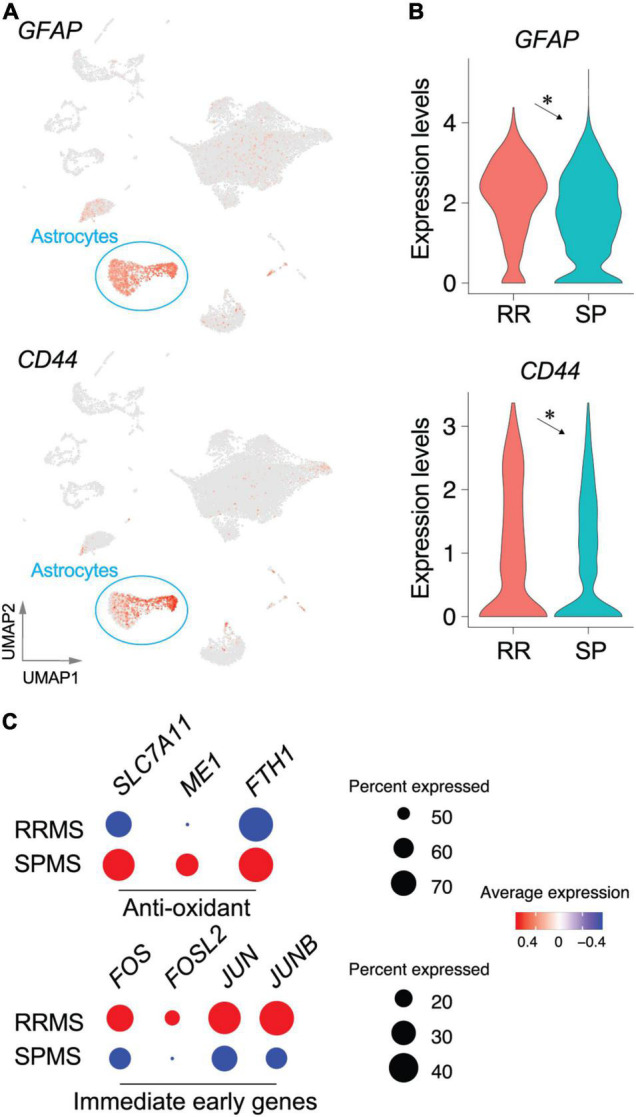
Transcriptomic changes in astrocyte populations between RRMS vs. SPMS. UMAP **(A)** and violin plots **(B)** of astrocyte marker genes. *, adjusted *p*-value < 0.05. **(C)** Dot plots of genes associated with antioxidant pathways and immediate early genes. Size and color indicate the CDRs and expression levels, respectively. Statistical results for DEGs, CDRs, and VAGs are provided in Supplementary Tables 4–6.

Astrocytes (RRMS 7.6%; SPMS 10.5% of total) encompassed 816 DEGs (Supplementary Table 3). RRMS astrocytes showed upregulation of marker genes for pan-reactive astrocytes including *GFAP* and *CD44*, as compared to SPMS (Figures 4A,B), suggesting that RRMS astrocytes were more reactive than SPMS astrocytes. Importantly, RRMS astrocytes expressed fewer genes associated with antioxidant pathways (*SLC7A11*, *ME1*, and *FTH1*) than SPMS astrocytes (Figure 4C). These results are also consistent with SPMS astrocytes reacting to severe oxidative stress that inhibits OPC maturation (French et al., 2009; Paintlia et al., 2011; Maus et al., 2015; Barateiro et al., 2016; Takase et al., 2018; Figure 3F). CDR analyses extracted 66 VAGs that included upregulation of *C3*, *SPP1* (encoding osteopontin that induces reactive astrocytes) (Kim et al., 2004; Moon and Shin, 2004; Gliem et al., 2015; Ikeshima-Kataoka et al., 2018), and *PFN1* [which modulates astrocytic morphology and motility (Schweinhuber et al., 2015)] in RRMS astrocytes (Supplementary Figure 3 and Supplementary Tables 5, 6). Collectively, these results implicated functional differences between RRMS (reactive phenotype) vs. SPMS (antioxidative phenotype). Of note, immediate-early genes (*FOS*, *FOSL2*, *JUN*, and *JUNB*) were found as DEGs in RRMS astrocytes over SPMS astrocytes (Figure 4C), which supported the previous identification of *immediate early astrocytes* (*ieAstrocytes)* that were identified in studies of EAE (an animal model of MS), and which increased in prevalence with disease severity (Groves et al., 2018).

### Functional Assessment of Single-Nucleus RNA Sequencing Gene Identification Using an Animal Model of Multiple Sclerosis

**FIGURE 5:**
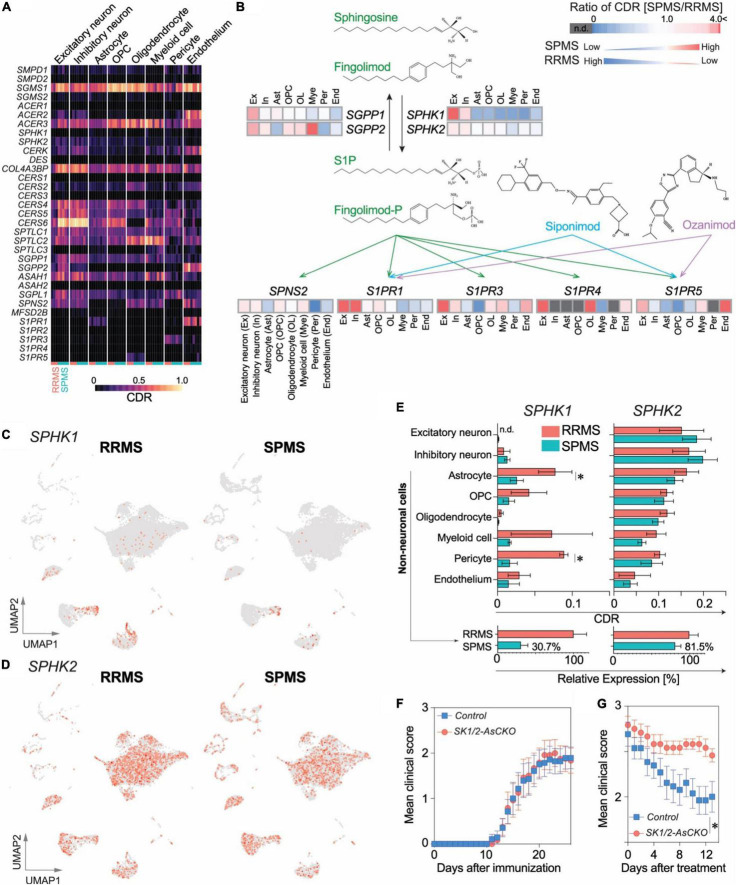
A potential role of CNS-derived sphingosine kinases (*SPHK1/2*) in fingolimod efficacy. **(A)** Heatmap of CDRs for sphingolipid pathway genes. **(B)** Comparison of CDR ratio between SPMS over RRMS in a pathway associated with the mechanism of action of S1P receptor modulators, fingolimod phosphate (fingolimod-P), siponimod, and ozanimod. UMAP plots of *SPHK1* **(C)** and *SPHK2* **(D)**. **(E)** *Top*: CDRs for *SPHK1* and *SPHK2*. *n* = 3 RRMS vs. 5 SPMS. *p < 0.05 by *t*-test. *Bottom*: Reductions in *SPHK1/2* expression in non-neuronal cell populations. **(F)** EAE disease course in Sphk1/2-AsCKO mice vs. control WT mice. *n* = 12 and 14 animals, respectively. **(G)** EAE clinical course of fingolimod-treated Sphk1/2-AsCKO mice vs. control WT mice. *n* = 12 and 13 animals, respectively. *p < 0.0001 by two-way ANOVA. Fingolimod treatment (1 mg/kg, p.o., q.d.) started from 18 days after immunization.

Fingolimod, a S1P receptor modulator that upon phosphorylation by sphingosine kinases, becomes an active agent (Brinkmann et al., 2010; Cohen and Chun, 2011; Kihara et al., 2015b; Kappos et al., 2018; Chun et al., 2019). It was the first oral agent approved to treat relapsing forms of MS; however, it failed to reach its primary endpoint in Phase 3 clinical trials in PPMS patients (Lublin et al., 2016). In contrast, another S1P receptor modulator, siponimod, successfully obtained approval for treatment of SPMS (Chun et al., 2020; Derfuss et al., 2020). Fingolimod’s lack of efficacy in progressive MS might be explained by alterations in sphingolipid pathway genes that affect fingolimod activity. Genes associated with fingolimod metabolism (*SPNS2*, *S1PR1*, *S1PR3*, *S1PR4*, *S1PR5*, *SPHK1* and *SGPP1*: Figures 5A,B and Supplementary Figure 4) were highly enriched in the top 30 CDR genes of “RRMS > SPMS” groups, markedly contrasting with their detection in “SPMS > RRMS” groups (Supplementary Figure 5). Most notably, sphingosine kinases (SPHK1 and SPHK2) that are required for fingolimod’s efficacy, but not for siponimod, were markedly reduced in SPMS as compared to RRMS (to approximately 30% for SPHK1 and approximately 80% for SPHK2, respectively, Figure 5E).

Astrocytes and pericytes that showed significant reduction in *SPHK1* expression (Figure 5E) were attractive targets to validate their functional significance in an animal model of MS. Considering the lower proportion of pericytes (∼1%) vs. astrocytes (∼10%) in the brain along with functional redundancies in sphingosine kinases and a pivotal role for S1P signaling involving astrocytes and fingolimod efficacy (Choi et al., 2011; Groves et al., 2018; Chun et al., 2019), astrocyte-specific Sphk1/2 conditional knockout (SK1/2-AsCKO) mice were utilized and challenged with EAE. The disease course in SK1/2-AsCKO mice was equivalent to their wild-type (WT) controls (Figure 5F), although S1P levels in EAE-induced SK1/2-AsCKO spinal cords (3.9 ± 2.1 pmol/mg tissue, *n* = 3) were significantly lower than those of WT spinal cords (11.3 ± 1.4 pmol/mg tissue, *n* = 4, *p* < 0.05 by *t*-test). These results suggested that astrocyte derived S1P was not involved in EAE pathogenesis. In contrast, fingolimod was not effective in EAE mice lacking astrocytic *Sphk1/2* genes (treatment, *p* < 0.0001; time, *p* = 0.003; interactions, *p* = 0.78; by two-way ANOVA, Figure 5G). These results implicated astrocyte SPHK1/2 in fingolimod efficacy and offer an explanation for its lack of efficacy in PPMS.

## Discussion

Multiple sclerosis is diagnostically characterized by detectable brain lesions, but global changes away from lesions, including gray matter and volumetric loss, are now accepted as part of MS disease manifestation (Bermel and Bakshi, 2006; Geurts et al., 2012). The use of normal-appearing, region-matched brain samples for snRNA-seq enabled generation of comparable cell clusters between conditions as demonstrated by highly overlapping UMAP layouts between RRMS vs. SPMS (Figure 1D) with approximately sixfold more transcripts per nucleus (5977 ± 735 vs. 5420 ± 725 transcripts/nucleus in RRMS vs. SPMS, respectively) than prior reports [1096 transcripts/nucleus (Jakel et al., 2019) and 2400 transcripts/nucleus (Schirmer et al., 2019)]. By contrast, previous snRNA-seq datasets focusing on MS lesions displayed non-overlapping distributions of cells (Jakel et al., 2019; Masuda et al., 2019; Schirmer et al., 2019). Our approach revealed pervasive transcriptomic changes with disease state that were independent of discernible lesion activity.

### Neuronal Dysfunction in Multiple Sclerosis Brains

Neurons (RRMS ∼3%; SPMS ∼17% of total) in our study were less represented than non-diseased brains (∼75% neurons) (Lake et al., 2018; Schirmer et al., 2019). Prior snRNA-seq studies of MS lesions compared to non-diseased control brains identified selective neuronal loss, severe cellular stress in OLs, increased reactive astrocytes, and microglial activation (Schirmer et al., 2019). These findings were also observed here along with additional features. First, neuronal dysfunction (loss of excitatory neuron marker genes) was found in normal-appearing RRMS brains as compared to SPMS brains, suggesting that RRMS brains had already commenced neuropathological changes irrespective of lesions (Figure 2). Second, the SPMS excitatory neurons involve axonal degeneration and impairment of neural network rewiring produced by upregulation of ion channels associated with channelopathy-like defects in SPMS (Kumar et al., 2016; Roostaei et al., 2016; Zhong et al., 2016).

### OPC Dysfunction in Secondary-Progressive MS

Approximately 5% of OPCs were identified from the normal-appearing MS brains, which was comparable to another study (7.8% in MS lesions vs. 6.0% in controls) (Schirmer et al., 2019). Upregulation of *SOX4* and *HES5* in SPMS OPCs, which inhibits OPC differentiation (Potzner et al., 2007; Braccioli et al., 2018; Munoz-Esquivel et al., 2019), along with downregulation of myelin formation-promoting genes, were identified (Figure 3). Moreover, genes that are essential for *de novo* sphingolipid synthesis including serine palmitoyltransferases (*SPTLC1/2/3*) and ceramide synthases (*CERS1/2/3/4/5*) were down-regulated in OLs and OPCs of SPMS as compared to RRMS, indicating dysfunctions in myelin synthesis with MS progression. Interestingly, *S1PR5* expression was significantly down-regulated in OLs and OPCs of SPMS over RRMS brains, which may impact second-generation S1PR modulators that bind to S1P_5_ in addition to S1P_1_. These results suggest that inhibitors targeting the SOX4-HES5 signaling pathway may be useful for promoting remyelination and delaying MS progression.

### Astrocyte Types in Multiple Sclerosis

A proposed classification of reactive astrocytes sub-divided into neurotoxic A1 astrocytes vs. helpful A2 astrocytes was based on gene expression patterns including immunolabeling for C3^+^, MX1^+^, or CFB^+^ of A1 astrocytes in MS lesions (Liddelow et al., 2017). The universality of this classification is unclear considering that low mRNA expression of A1 markers is found in MS lesions (Schirmer et al., 2019). Moreover, A1 or A2 reactive astrocyte genes were rarely found in DEGs or CDRs in the present study, except for *C3* that was more enriched in microglia rather than astrocytes (Supplementary Figure 6). A1 astrocyte formation has been proposed to require three microglia-derived factors [IL1α, TNF-α, and C1q (Liddelow et al., 2017)] that were not identified in a prior study (Schirmer et al., 2019). However, considerable C1q gene expression (*C1QA*, *C1QB*, and *C1QC*) in microglia, along with minor expression of *IL1A* and *TNF*, was observed here (Supplementary Figure 5). The expression differences of microglia-derived A1 factor genes between prior studies and the current work may reflect the approximately sixfold increased numbers of genes identified in each cell. Collectively, the A1/A2 classification was only partially supported by some CDRs in A1 gene sets compared to A2 gene sets, indicating a need for further study in assessing MS pathogenesis related to A1/A2 astrocytes.

An alternative and not mutually exclusive astrocyte classification is *ieAstrocytes* [immediate early astrocytes (Groves et al., 2018)] that were identified by unbiased *in vivo* c-Fos activity screen in EAE spinal cords. Importantly, *ieAstrocytes* formation is highly correlated with EAE severity and is blocked by S1P_1_ inhibition or deletion (Groves et al., 2018), which identified potential involvement of *ieAstrocytes* in MS pathology. Transcriptomic signature of *ieAstrocytes* was supported by the present study (Figure 4) through *FOS* (c-Fos)-expressing astrocytes documented in normal-appearing MS brains, along with upregulation in RRMS over SPMS (Figure 4C). Datasets from prior study also supported this concept through *FOS* upregulation in astrocytes of MS lesions (Schirmer et al., 2019). The current report indicates that *ieAstrocyte* characteristics extend beyond lesions to normal-appearing regions of the MS brain (Figure 4C).

### Implications for the Efficacy of Sphingosine1-Phosphate Receptor Modulators

Protective effect of fingolimod in EAE was abolished in global *Sphk2*-deficient mice (Imeri et al., 2016), which clearly demonstrates the requirement of SPHK activity for the fingolimod’s efficacy. A notable finding here was the perturbation of sphingolipid metabolic genes, particularly the reduction of *SPHK1/2*-expressing cells in the SPMS brain (Figure 5). This was consistent with a prior study (Schirmer et al., 2019) where *SPHK1/2* expression was lower in chronic inactive lesions [predominately in SPMS (Frischer et al., 2015)] than acute/chronic active lesions [found more often in RRMS (Frischer et al., 2015)]. Reduction of *SPHK1/2*-expressing cells in the CNS may, in part, account for the clinical trial failure of fingolimod for PPMS treatment (Lublin et al., 2016). In addition, the S1P transporter SPNS2 (spinster 2), which transports not only S1P but also fingolimod phosphate (Hisano et al., 2011), was also significantly reduced in SPMS astrocytes as compared to RRMS astrocytes (Figure 5 and Supplementary Table 5). These results along with a prior report on fingolimod accumulation in the CNS (Foster et al., 2007) propose that fingolimod activity appears to involve local metabolic steps in astrocytes including SPHK1/2-mediated fingolimod phosphorylation and its secretion *via* transporters. We recently discovered that astrocytic S1P_1_ inhibition results in neuroinflammatory resolution *via* modulating vitamin B_12_ and type I interferon systems (Jonnalagadda et al., 2022). Thus, the requirement of astrocyte S1P_1_ on EAE development and fingolimod efficacy (Choi et al., 2011; Groves et al., 2018) further support the local fingolimod actions in the CNS (Chun et al., 2019; Kihara, 2019). The mechanisms underlying the reduction of SPHK-expressing CNS cells in MS brains requires further study but may represent a novel area for therapeutic intervention targeting astrocytes. Downregulation of SPHK1 mRNA expression in SPMS astrocytes might be explained by the reduction of protein kinase C (PKC; gene name, *PRKCA*: Supplementary Table 5) that induces *SPHK1* expression (Sobue et al., 2008). Similar mechanisms may contribute to reduced expression of sphingolipid metabolism and S1PR genes in SPMS brains, which need to be further investigated. In contrast to fingolimod, next generation S1P receptor modulators do not require phosphorylation by SPHKs for their activity (Kihara et al., 2014, 2015b; Chun et al., 2019; Kihara, 2019), explaining the efficacy of at least one tested agent, siponimod, in progressive MS (Kappos et al., 2018).

Taken together, the current snRNA-seq dataset complements prior snRNA-seq MS lesion datasets to reveal neural dysfunction within normal-appearing MS-affected brains. The perturbations of sphingolipid signaling pathways may help to explain the lack of efficacy for fingolimod in PPMS and implicates non-prodrug strategies to impact sphingolipid signaling in progressive CNS disorders.

## Data Availability Statement

The datasets presented in this study can be found in online repositories. The names of the repository/repositories and accession number(s) can be found below: https://www.ncbi.nlm.nih.gov/geo/, GSE17959.

## Ethics Statement

The animal study was reviewd and approved by the Institutional Animal Care and Use Committee of the Sanford Burnham Prebys Medical Discovery Institute.

## Author Contributions

YK, YZ, DJ, WR, CP, BS, and RR performed the experiments, analyzed the data, and wrote the manuscript. RD and BT collected the human MS brains. JC conceived of the project and wrote the manuscript with the co-authors. All authors contributed to the final version of the manuscript.

## Author Disclaimer

The content is solely the responsibility of the authors and does not necessarily represent the official views of the National Institutes of Health.

## Conflict of Interest

JC received honoraria, consulting fees, and research grants from Novartis, Bristol-Myers Squibb (Celgene), Biogen, and Janssen Pharmaceuticals. The remaining authors declare that the research was conducted in the absence of any commercial or financial relationships that could be construed as a potential conflict of interest. The handling editor declared a past co-authorship with one of the authors, JC.

## Publisher’s Note

All claims expressed in this article are solely those of the authors and do not necessarily represent those of their affiliated organizations, or those of the publisher, the editors and the reviewers. Any product that may be evaluated in this article, or claim that may be made by its manufacturer, is not guaranteed or endorsed by the publisher.

## Footnotes

1
https://reactome.org/

2
https://www.ncbi.nlm.nih.gov/geo/
