# Supplementary material for: Single-Nucleus RNA-seq of Normal-Appearing Brain Regions in Relapsing-Remitting vs. Secondary Progressive Multiple Sclerosis: Implications for the Efficacy of Fingolimod
Source: Front Cell Neurosci. 2022 Jun 17;16:918041. doi: 10.3389/fncel.2022.918041 (PMC9247150; doi:10.3389/fncel.2022.918041)
Supplement: Supplementary file 1 [file Data_Sheet_1.docx]

**Supplemental Data**

**Single-nucleus RNA-seq of normal-appearing brain regions in relapsing-remitting vs. secondary progressive multiple sclerosis: Implications for the efficacy of fingolimod**

**Authors:** Yasuyuki Kihara^1,*^, Yunjiao Zhu^1,4*^, Deepa Jonnalagadda^1,*^, William Romanow^1^, Carter Palmer^1,3^, Benjamin Siddoway^1,5^, Richard Rivera^1^, Ranjan Dutta^2^, Bruce D. Trapp^2^, and Jerold Chun^1,#^

**Supplemental Figures provided as Supplemental_Figures.pdf**

- Supplemental Figure S1. Heatmap of cell type-specific gene expression.
- Supplemental Figure S2. Transcriptomic signatures in other cell types.
- Supplemental Figure S3. Comparison between DEGs vs. CDR genes in each cell type.
- Supplemental Figure S4. Top 30 CDR genes in the sphingolipid pathway.
- Supplemental Figure S5. Pan/A1/A2-specific genes.

**Supplemental Tables provided as Supplemental_Tables.xlsx**

- Supplemental Table S1: Sample information
- Supplemental Table S2: Stats for snRNA-seq results
- Supplemental Table S3: Proportion of cell types and their marker genes
- Supplemental Table S4: DEGs in each cell type
- Supplemental Table S5: Genes identified by CDR analyses and VAGs in each cell type
- Supplemental Table S6: VAGs in each cell type
- Supplemental Table S7: Reactome pathway analysis of DEGs found in excitatory neurons
- Supplemental Table S8: Reactome pathway analysis of VAGs found in OLs
- Supplemental Table S9: Reactome pathway analysis of VAGs found in OPCs


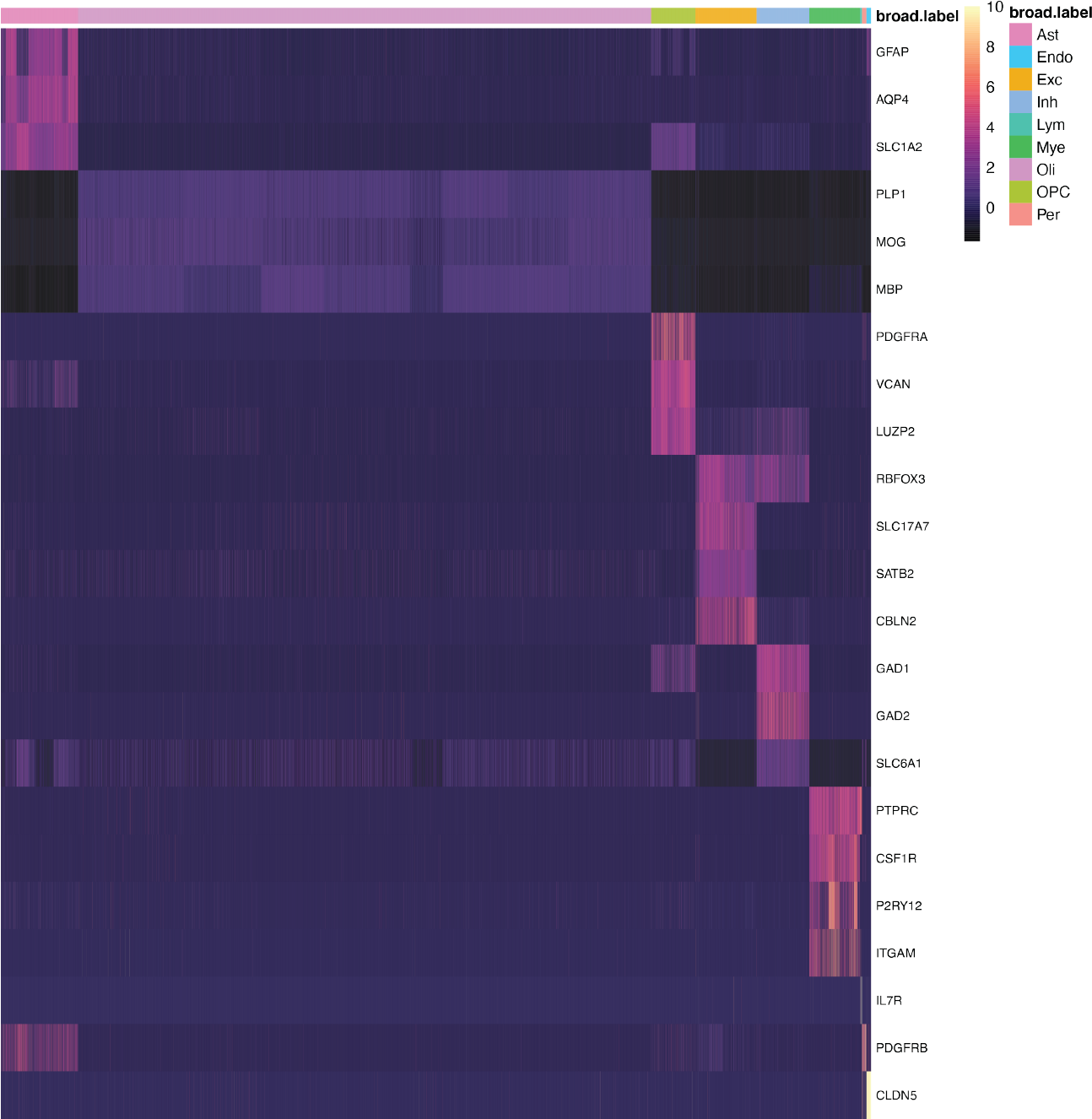


**Figure S1. A Heatmap of cell type-specific gene expression.**

***Microglia activation genes are increased in RRMS over SPMS***

Myeloid cells (RRMS 7.3%; SPMS 10.5% of total) (**Fig. 1E and Supplemental Table S3**), were associated with decreased expression of microglial core genes (*P2RY12*, *P2RY13*, and *CX3CR1*) in RRMS over SPMS (**Supplemental Figure S2**). Activation marker genes for myeloid cells (*CD68*, *CD74*, *FTL*, *APOE*, and *SPP1*) were higher in RRMS than SPMS (**Supplemental Figure S2**), indicating the increased microglial activation in RRMS brains.

***SPMS lymphocytes and astrocytes are possible sources of a SPMS marker gene, MALAT1***

Pericytes (~0.5% of total), endothelial cells (~0.6% of total), and lymphocytes (~0.2% of total,) were detected (**Fig. 1E and Supplemental Table S3**), which expressed fewer DEGs (8, 35 and 1 DEGs, respectively) than other cell types. Lymphocyte clusters in normal-appearing MS brains (~0.20% of total) were generally comparable to those in prior reports (~0.6% in MS brains as compared to 0% in control non-diseased brains (Schirmer et al., 2019)). The only DEG found in lymphocytes (**Supplemental Figure S2**) was long non-coding *MALAT1* that was elevated in SPMS (**Supplemental Figure S2**), supporting a previous report identifying *MALAT1* as a potential biomarker for SPMS diagnosis (Shaker et al., 2019). Because the upregulation of *MALAT1* was also found in astrocytes (**Supplemental Figure S2**), both lymphocytes and astrocytes might be possible sources of increased *MALAT1* expression in SPMS.

**
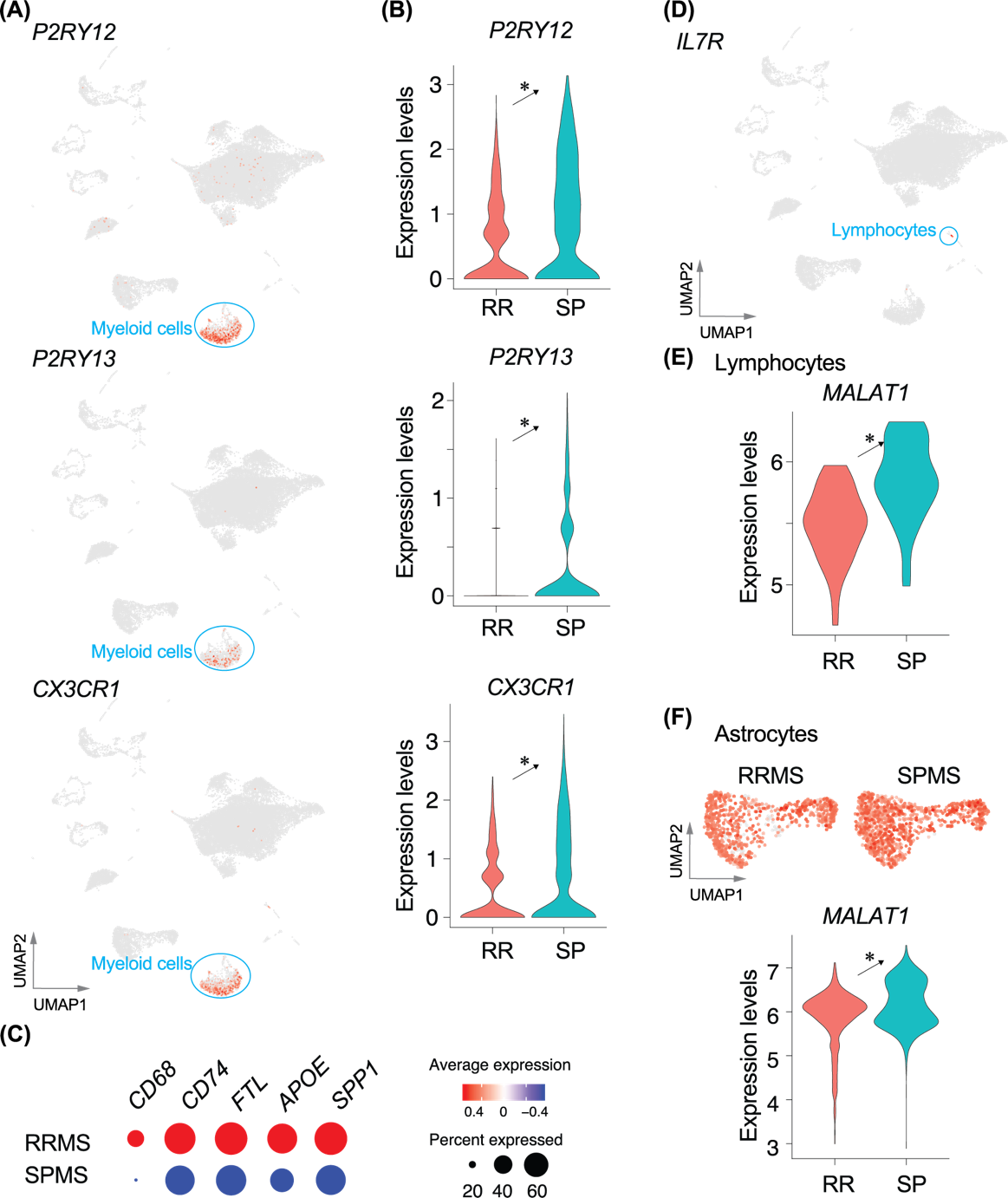
**

**Figure S2. Transcriptomic signatures in other cell types.** (**A and B**) UMAP (A) and violin plots (B) of microglial marker genes. *, adjusted p value < 0.05. (**C**) Dot plots of activation marker genes for myeloid cells. Size and color indicate the CDRs and expression levels, respectively. (**D and E**) UMAP of lymphocyte marker gene IL7R (D), and violin plot of *MALAT1* (E). (**F**) UMAP and violin plot of *MALAT1* expression in astrocytes. *, adjusted p value < 0.05. Statistical results for DEGs, CDRs and VAGs are provided in **Supplemental Table**.


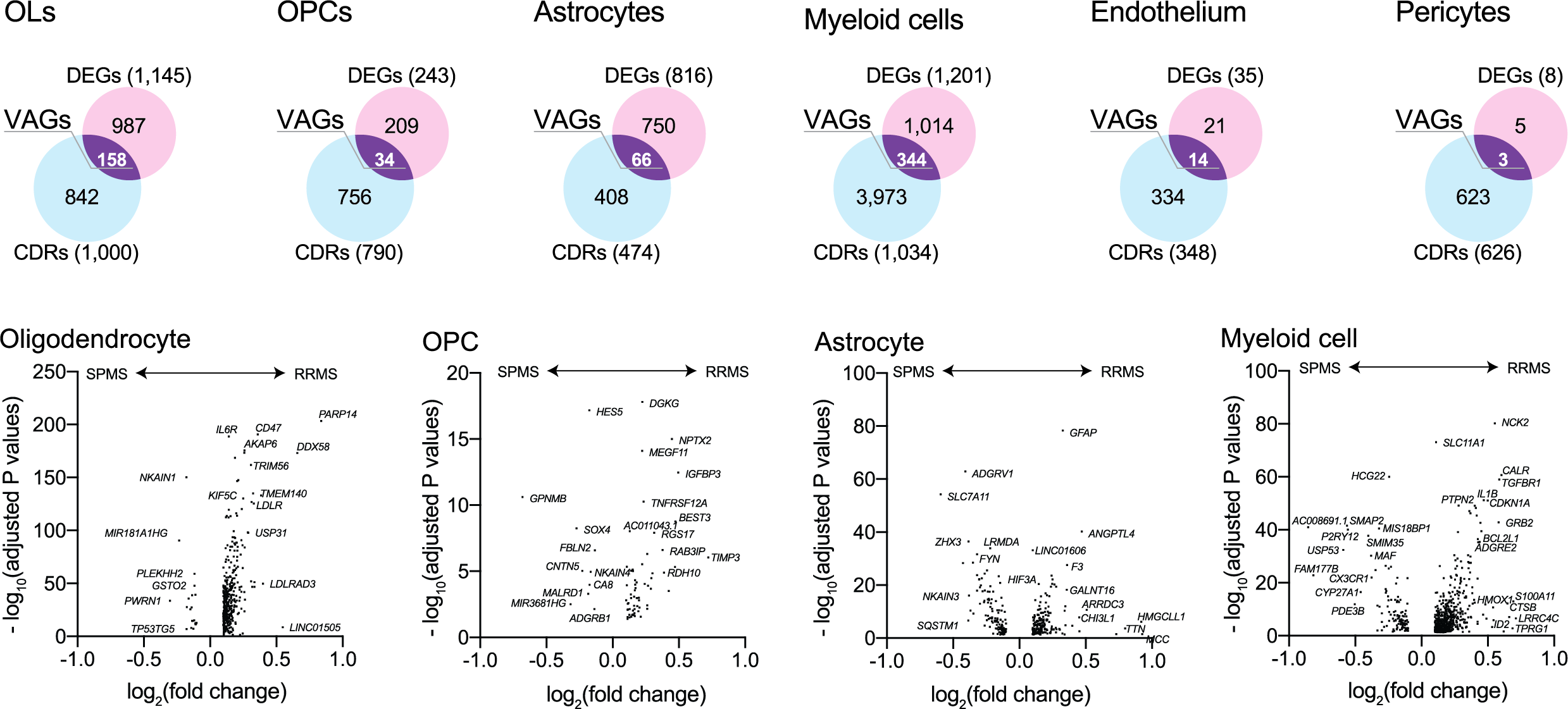


**Figure S3. Comparison between DEGs vs. CDR genes in each cell type.** (Top) Venn diagrams between DEGs and CDRs. The intersections are VAGs. (Bottom) Plots for VAGs in oligodendrocytes, OPCs, astrocytes, and myeloid cells.


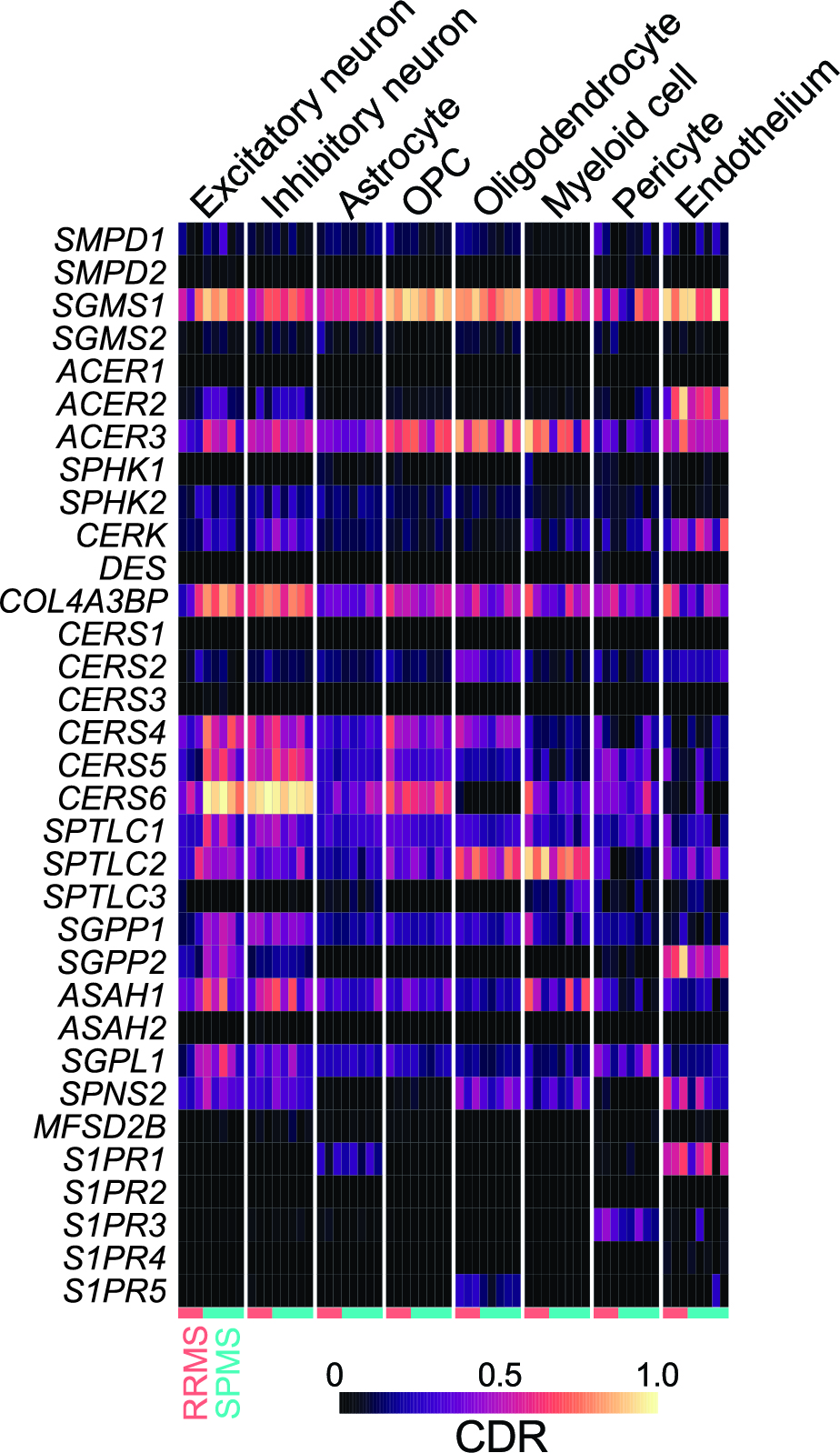


**Figure S4. Enlarged heatmap of CDRs for sphingolipid pathway genes.**


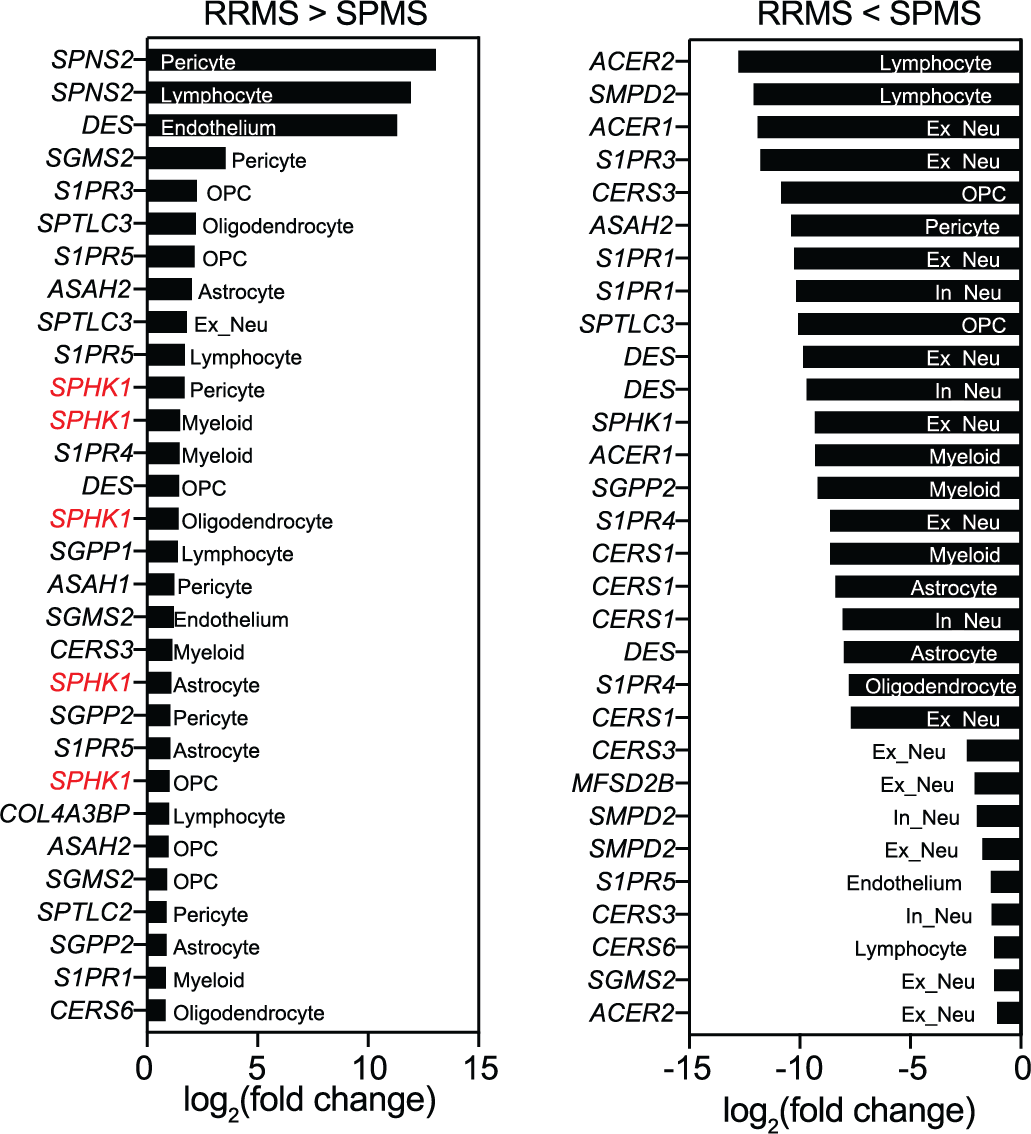


**Figure S5. Top 30 CDR genes in the sphingolipid pathway.**


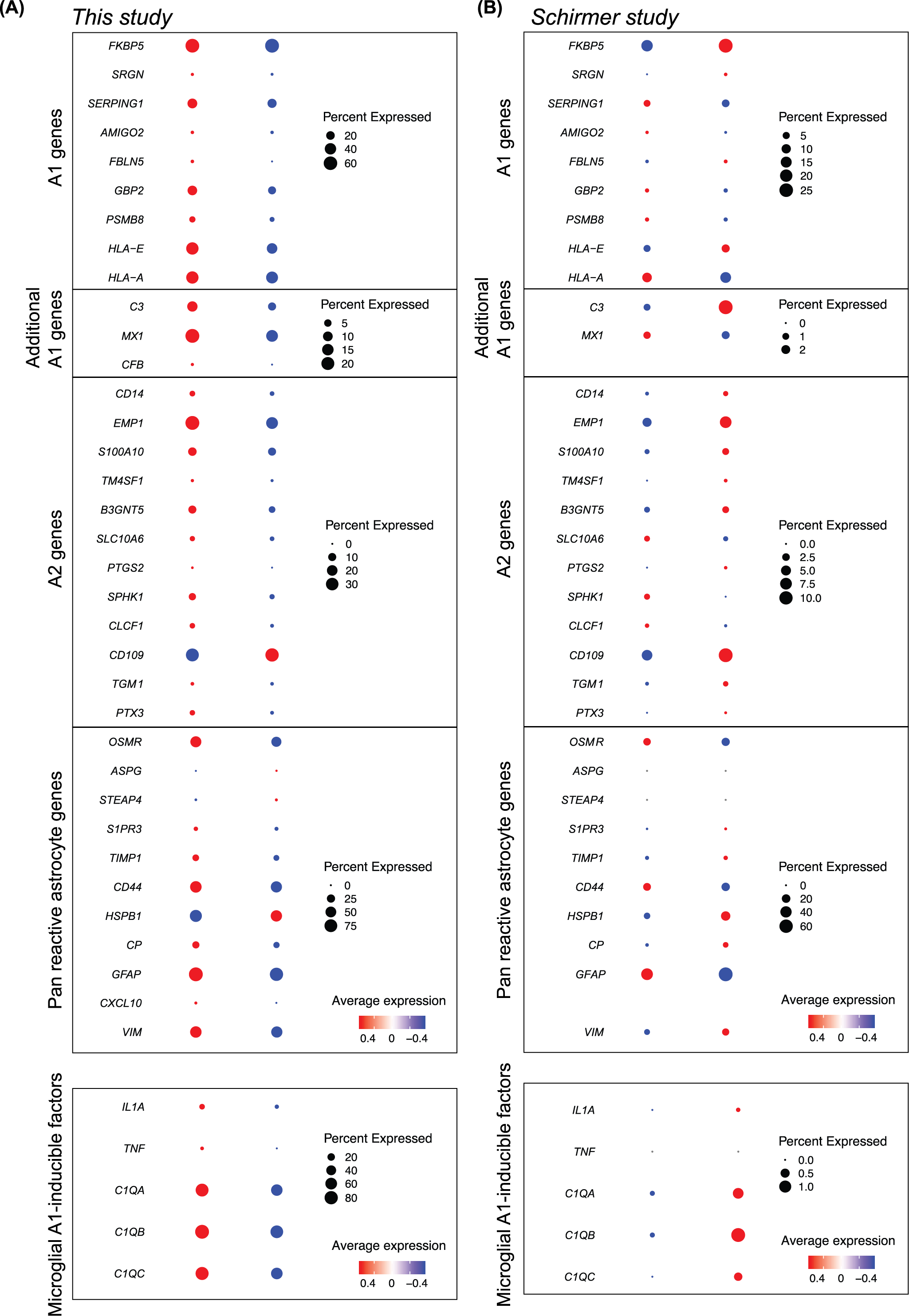


**Figure S6. Astrocytic gene expression associated with A1/A2/Pan reactive astrocytes and expression of microglial A1-inducible factors.**
